# Expanded universal carrier screening and its implementation within a publicly funded healthcare service

**DOI:** 10.1007/s12687-019-00443-6

**Published:** 2019-12-11

**Authors:** Charlotte A. Rowe, Caroline F Wright

**Affiliations:** 1 University of Exeter, St Luke’s Campus, 79 Heavitree Rd, Exeter, EX1 1TX UK; 2 Post Graduate Centre, Royal Cornwall Hospitals NHS Trust, Treliske, Truro, Cornwall TR1 3LQ UK; 3 Institute of Biomedical and Clinical Science, College of Medicine and Health, University of Exeter, RILD Building, RD&E, Barrack Road, Exeter, EX2 5DW UK

**Keywords:** Carrier screening, Genome sequencing, Universal, Expanded

## Abstract

Carrier screening, a well-established clinical initiative, has been slow to take advantage of the new possibilities offered by high-throughput next generation sequencing technologies. There is evidence of significant benefit in expanding carrier screening to include multiple autosomal recessive conditions and offering a ‘universal’ carrier screen that could be used for a pan-ethnic population. However, the challenges of implementing such a programme and the difficulties of demonstrating efficacy worthy of public health investment are significant barriers. In order for such a programme to be successful, it would need to be applicable and acceptable to the population, which may be ethnically and culturally diverse. There are significant practical and ethical implications associated with determining which variants, genes and conditions to include whilst maintaining adequate sensitivity and accuracy. Although preconception screening would maximise the potential benefits from universal carrier screening, the resource implications of different modes of delivery need to be carefully evaluated and balanced against maximising reproductive autonomy and ensuring equity of access. Currently, although a number of existing initiatives are increasing access to carrier screening, there is insufficient evidence to inform the development of a publicly funded, expanded, universal carrier screening programme that would justify investment over other healthcare interventions.

## Introduction

Carrier screening for genetic conditions historically involves screening asymptomatic individuals and couples within a high-risk population for heterozygous carriers of specific autosomal recessive (AR) conditions. Carrier screening initiatives began in the 1970s with screening Ashkenazi Jewish populations for Tay-Sachs disease (TSD) (Kaback 2001), enabling carriers to make reproductive decisions based on a quantified risk of having an affected child. Technological advances over the last decade have now made genome-wide sequencing (GWS) affordable, potentially enabling carrier screening to be ‘expanded’ to include more conditions (van der Hout et al. 2016) and 'universal' to be offered beyond high-risk groups (Lazarin and Haque 2016). The integration of such a programme into a publicly funded healthcare service, such as the National Health Service (NHS) in the UK, would facilitate widespread access to potential benefits of the genomic era. The aim of this review is to evaluate the existing evidence for expanded universal carrier screening (EUCS) (van der Hout et al. 2016) programmes, and to appraise the potential benefits and challenges of implementing such a programme within a publicly funded healthcare system serving an ethnically diverse population.

## Methods

**Fig. 1:**
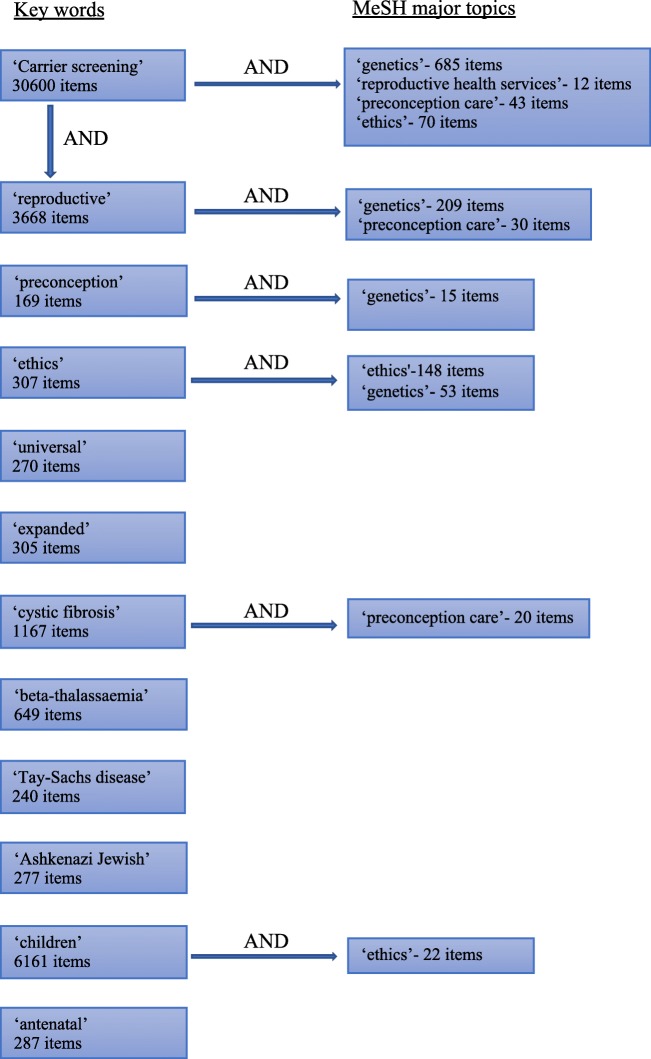
Results generated using specific keywords and MeSH major topics. The additional use of MeSH major topics achieved results of greater relevance. Items shown on initial search on 3 August 2018

Keywords and MeSH major topics used to search PubMed for relevant studies and review papers are shown in Fig. 1. Non-English language papers were excluded. Titles were screened for relevance, and selected abstracts and discussion were reviewed. Scrutiny of methods and supplementary material was carried out for studies discussed in depth. Review papers were used to gain an overview of topics and to identify relevant studies that may have been missed on PubMed searches. The UK Government (https://www.gov.uk) and NHS digital (https://digital.nhs.uk) websites were utilised to search for up-to-date epidemiological data in the UK.

## Background

### The impact of AR conditions

EURODIS estimates that 6–8% of the population are affected by a rare disease (EURODIS Rare Diseases Europe 2018) and AR conditions make up a significant proportion of this. The burden of AR conditions varies substantially between populations due to geographical isolation and differing levels of consanguinity (Antonarakis 2019). It has been estimated that, of 7028 diseases with suspected Mendelian inheritance, 1139 are recessive (Bell et al. 2011). However, these are likely to be underestimates and the true prevalence of AR conditions is still unknown (Antonarakis 2019). Appraising the impact of AR conditions is also challenging and recent high-impact studies (Bell et al. 2011; Hogan et al. 2018) are still quoting old data; that Mendelian diseases account for 20% of infant mortality (Costa et al. 1985) and between 10 and 34% of infant hospitalisations (Kumar et al. 2001; McCandless et al. 2004). The majority of AR conditions have significant impact on not only the individual’s health and quality of life (Dudding et al. 2000; Cousens et al. 2010; Lew et al. 2012; Jamieson et al. 2014) but also that of their families. Parents and families of children with health disabilities experience significant financial and psychological stress (Goudie et al. 2014) and often avoid having another child with the same condition (Dudding et al. 2000).

### Single-condition versus expanded carrier screening

In the UK, where a publicly funded national healthcare system serves a multi-ethnic and multi-cultural population, carrier screening is offered universally for beta-thalassaemia and sickle cell anaemia as part of routine antenatal care (Public Health England 2017–2018). Screening for other AR conditions, such as Cystic Fibrosis (CF) and TSD, is only offered to certain high-risk groups based on ancestry and family history (UK Genetic Testing Network) (Table 1). Carrier screening programmes worldwide respond to the needs, ethnicity, and culture of specific populations (Cao et al. 1981, 1985; Angastiniotis and Hadjiminas 1981; Cowan 2009; Scriver et al. 1984; Mitchell et al. 1996; Greengross et al. 1999; Tarazi et al. 2007; Alhamdan et al. 2007; Stafler et al. 2016; Castellani et al. 2015; Cunningham and Marshall 1998; Lew et al. 2012). Most screen for a single condition and have shown remarkable reductions in the frequency of affected births (Table 2). Particular successes are seen for beta-thalassaemia (Cao et al. 1981,^1985^; Tarazi et al. 2007; Greengross et al. 1999; Angastiniotis and Hadjiminas 1981; Cowan 2009; Scriver et al. 1984; Mitchell et al. 1996; Alhamdan et al. 2007) and TSD (Mitchell et al. 1996; Lew et al. 2012) with up to 95% decrease in the frequency of affected births. However, it should be noted that screening for beta-thalassaemia, sickle cell, and until recently TSD uses haematological and biochemical markers, respectively. These programmes have therefore avoided many of the problems of DNA-based carrier screening, which will be discussed.

**Table 1:** Current carrier screening programmes in the UK. Table compiled using information from NHS Sickle Cell and Thalassaemia Screening Programme (Public Health England 2017–2018) and UK Genetic Testing Network (2018). *CF* cystic fibrosis, *TSD* Tay-Sachs disease

Condition	Target population	Details of test	Timing of test
ß-Thalassemia	All pregnant women	Carrier status is determined based on red blood cell indices (not DNA). If the woman is found to be a carrier, screening is then offered to the baby’s biological father.	Antenatal
Sickle cell disease	All pregnant women		Antenatal
Cystic fibrosis	- Patients with a family history of CF	Varies from specific variants to sequencing of the whole coding region of the *CFTR* gene	Preconception
	- Partner is a known carrier of CF		Antenatal
	- Close consanguineous couple AND from ethnic group with higher carrier frequency		Opportunistic (100,000 Genomes project)
	- Can opt-in to ‘additional findings’ if eligible and taking part in the 100 K Genomes project.		
Tay-Sachs disease	- Patients of Ashkenazi Jewish ancestry	Uses biochemical assays to identify carriers. Further characterization of carriers with DNA testing	Preconception
	- Patients with a family history of TSD		Antenatal

**Table 2:** A comparison of different worldwide screening programs and their outcomes. *S* during school years, *PM* premarital, *PC* preconception, *A* antenatal, *BT* beta-thalassaemia, *CF* cystic fibrosis, *TSD* Tay-Sachs disease

Authors	Country	Condition	Overview	Timing of screening	Implementation	Pre-screening education?	Counselling offered for carriers?	% decrease in frequency of affected births
Cao et al. (1985); Cao et al. (1981)	Sardinia, Italy	BT	Voluntary, universal screening programme since 1975	PM or A	Takes place in community and hospital settings	Yes	Yes	95%
Angastiniotis and Hadjiminas (1981); Cowan (2009)	Cyprus	BT	Quasi-mandatory screening amount Greek-Cypriots and mandatory government screening among Turkish Cypriots. Universal screening since 1973	PM, PC, or A	Screening takes place after marriage intention is registered	Yes	Yes	85%
Scriver et al. (1984); Mitchell et al. (1996)	Montreal, Canada	BT	Voluntary screening program of high-risk populations 1979–1992	S, PM	Took place in schools and community centres	Yes	Yes	94%
Greengross et al. (1999)	London, England	BT	Voluntary, universal screening program since 1985	A	Screening offered during routine antenatal care	No	Yes	80%
Tarazi et al. (2007)	Palestinian District, Gaza Strip	BT	Mandatory screening. Universal since 2000	PM	Screening takes place after marriage intention is registered	Yes	Yes	75–80%
AlHamdan et al. (2007)	Saudi Arabia	BT	Mandatory screening. Universal since 2003	PM	Screening takes place after marriage intention is registered	No	Yes	Minimal change (89% of carrier couples still married and terminations not widely practiced)
Stafler et al. (2016)	Israel	CF	Voluntary, universal screening since 2008	A	Offered during routine antenatal care	Yes	Yes	> 50%
Castellani et al. (2015)	North-eastern Italy	CF	Voluntary, universal screening since 1994	PC or A	Offered in GP clinics and by gynaecologists	Yes	Yes	15% (annual)
Cunningham and Marshall (1998)	Edinburgh, Scotland	CF	Voluntary, universal screening since 1990	A	Antenatal-offered during routine antenatal care	Not formally	Yes	65%
Lew et al. (2012)	Sydney and Melbourne, Australia	TSD	Voluntary, targeted to Jewish population. Started in 1995	S	Took place in Jewish senior high schools. Funding by Jewish community	Yes	Yes	50%
Mitchell et al. (1996)	Quebec, Canada	TSD	Voluntary screening program of high-risk populations 1979–1992	S, PM	Took place in schools and community centres	Yes	Yes	90%

Inclusion criteria: (1) studies objectively evaluating the impact of single conditions carrier screening on a specific population which reported on the timing of screening and on education and counselling, (2) publication date 1980–present, (3) all geographical locations, (4) English language. Outcome measures: frequency of affected births before and after intervention

Next-generation sequencing technologies (Metzker 2010) enable genetic variants across multiple genes to be tested simultaneously in a cost-efficient fashion whilst maintaining accuracy comparable to single-gene tests (Srinivasan et al. 2010). Single-condition carrier screening successes have fuelled the development of expanded gene panels (ACOG 2009; Gross et al. 2008; Ioannou et al. 2010; Scott et al. 2010; Shao et al. 2015), e.g. in Victoria, Australia, where carrier screening for seven conditions is offered to students in Jewish high schools (Ioannou et al. 2010). These programmes have been well received (Shao et al. 2015; Scott et al. 2010) and suggest that expanded carrier screening could provide a cost-effective option that identifies more carriers and thus increases the number of people who could benefit.

### Universal versus ancestry-based carrier screening

Carrier screening has traditionally been targeted towards specific high-risk groups based primarily on ethnicity, but there are convincing arguments (Box 1) to move towards a universal screening programme, offered to everyone, regardless of ancestry. In the UK, CF is the most common AR condition in the Caucasian population, with an estimated carrier frequency of 1 in 25 (Massie and Delatycki 2013); however, testing is offered only when certain criteria are met (Table 1) and relies on self-reported risk or recognition of high-risk couples by health professionals. It has long been argued that this method misses many carriers (Boulton et al. 1996; Williamson 1993). An estimated 94% of newborns with CF are born to families with no family history (McClaren et al. 2011) and two-thirds of families would elect to avoid having another child with CF (Dudding et al. 2000). This, arguably, demonstrates considerable inadequacy of the current programme.

**Table:** 

TARGETED screening (only offered to those with known pre-existing risk i.e. ancestry)	
Advantages:	
- Cheaper (as fewer individuals are screened)	
- Higher pick-up rate in higher risk populations	
Disadvantages:	
- Carriers likely to be missed	
- Contributes to ethnic stigmatisation	
UNIVERSAL screening (screening offered to everyone, regardless of pre-existing risk)	
Advantages:	
- Carriers less likely to be missed	
- Avoids ethnic stigmatisation	
- Likely lead to an increase in testing within minority groups due to increased availability and decreased stigmatisation.	
Disadvantages:	
- More expensive	
- Requires upskilling of health professionals	
- Administratively challenging to deliver	

The UK population consists of multiple ethnicities and the number of people reporting ‘mixed racial ancestry’ is rising (Office for National Statistics 2011). This increases the likelihood of missing carriers if screening is ancestry-based. Lazarin et al. found up to 50% of carriers did not fit the American College of Medical Genetics and Genomics (ACMG) screening criteria for 10 different AR conditions (Table 3), such as being from ‘low-risk’ ethnic groups or an absence of family history (Lazarin et al. 2013). Advantages of universal screening include the abolishment of ethnic or racial factors, reducing stigmatisation and removing the onus on patients or clinicians to recognise risk. However, universal screening would increase costs and complicate genetic variant analysis across different laboratories. There would be a need to ensure adequate accuracy and sensitivity across the whole population.

**Table 3:** Autosomal recessive conditions where testing is targeted to specific ethnic groups as per ACMG and ACOG guidance. Significant numbers of carriers were outside the target group and thus would have been missed. Table adapted from Lazarin et al. (2013)

Disease	Population targets	Total carriers	Non-targeted carriers	% missed carriers
Sickle cell disease	African-American	145	56	38.6
Beta-thalassemia	African- American	163	72	44.2
	Southern European			
	South Asian			
	Southeast Asian			
Canavan disease	Ashkenazi Jews	71	28	39.4
Familial dysautonomia	Ashkenazi Jews	76	20	26.3
Tay-Sachs disease	Ashkenazi Jews	151	61	40.4
Fanconi Anaemia Group C	Ashkenazi Jews	44	20	45.5
Niemann–Pick type A	Ashkenazi Jews	33	8	24.2
Bloom syndrome	Ashkenazi Jews	35	12	34.3
Mucolipidosis IV	Ashkenazi Jews	36	18	50.0
Gaucher disease	Ashkenazi Jews	280	138	49.3

## Design and implementation strategies

### Gene panel design

EUCS panels are already available (Scott et al. 2010); however, there are considerable differences in panel composition between laboratories (Hoffman et al. 2014) with varying opinions on which conditions should be included. Whilst many individual AR conditions are rare, collectively, they are thought to account for significant mortality and morbidity (Srinivasan et al. 2010). Biotechnology company Myriad (previously Counsyl) (offering a for-profit carrier screening service) reported that the sum of carrier frequencies of rarer AR conditions exceeds that of more common ones (Srinivasan et al. 2010). Some argue that large-scale EUCS for many rare AR and X-linked conditions could have a greater impact in reducing mortality and morbidity than just screening for common AR conditions (Lazarin and Haque 2016). However, how many of the > 1000 known AR conditions should be included in an expanded carrier screening panel is a topic of considerable debate, with the ideal outcome being to identify as many carrier couples as possible whilst balancing the risk of harms and cost implications.

**Fig. 2:**
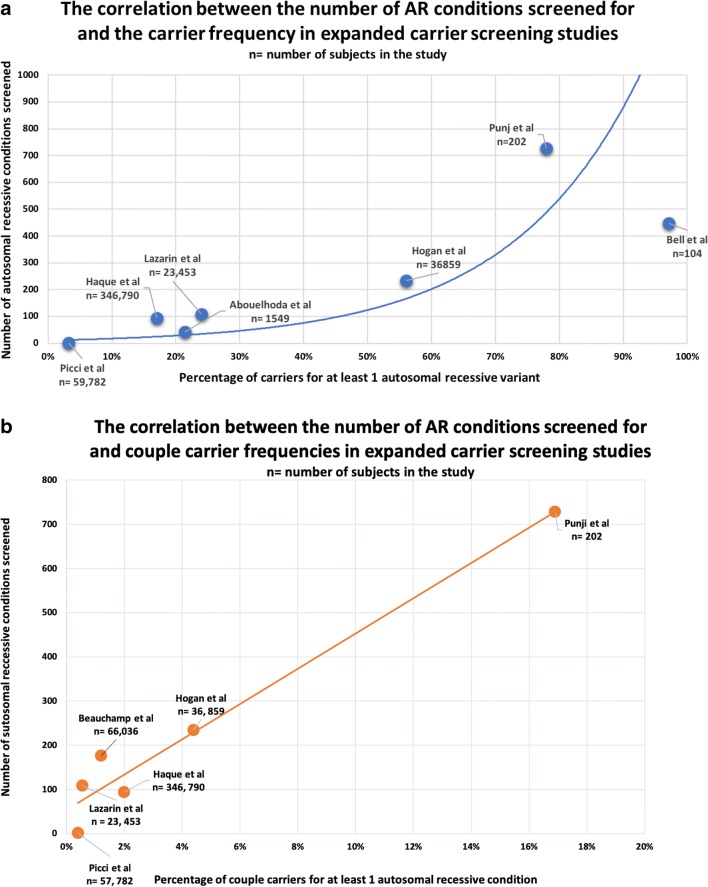
Plot of percentage of **a** individuals and **b** couples found to be carriers for at least one AR variant increasing in relation to the number of autosomal recessive conditions screened for. The exponential line of best fit in **a** suggests there would be a saturation point reached where the percentage of carriers would plateau. In **b**, the line of best fit appears to be linear but there is significant clustering of data and Punj et al. is a potential outlier. Data from following studies: Picci et al., Haque et al., Abouelhoda et al., Lazarin et al. (2013), Hogan et al. (2018), Punj et al. (2018), Bell et al. (2011), and Beauchamp et al. (2019)

Predictably, the more AR conditions included, the more carriers and carrier couples will be identified (Fig. 2a, b, Table 4). Indeed, every individual is likely to be a carrier for at least one AR condition. However, estimates from existing expanded carrier screening studies may not be applicable to the general population where carrier frequencies for individual AR conditions are generally low, and identifying carrier couples for the majority of AR conditions will likely be rare (Antonarakis 2019). Bell et al. showed very high carrier frequencies, likely because 73% of the study population were already known to be carriers or affected by severe, childhood recessive disorders (Bell et al. 2011). Similarly, Punj et al. found a carrier couple frequency of 16.9%, but 3% of the study population were already known carriers for CF and screening of male partners only took place after a woman had received a positive result (Punj et al. 2018). Haque et al. screened the largest number of subjects which were a realistic representation of the general population and found the overall risk of a ‘hypothetical’ foetus affected by one of the 94 conditions on the screening panel was between 0.09–0.3% (variability depended on ethnicity) (Haque et al. 2016). For comparison, the background risk of a Caucasian couple (unknown carrier status) having a child affected by CF is 0.04% (Massie and Delatycki 2013). Therefore, the additional 93 conditions could potentially prevent up to 1 in 384 births being affected by an AR condition.

**Table 4:** A comparison of carrier screening studies, the approach used, the number of AR conditions/variants screened for and carrier/carrier couple frequencies. *n* number of study subjects, *nAR* number of autosomal recessive conditions included in the panel, *MAR* medically assisted reproduction, *CF* cystic fibrosis

Study	Overview	*n*	nAR	Genes/variants	Approach	% of carriers for at least 1 variant	% of couple carriers	Other findings/comments
Lazarin et al. (2013)	Large-scale expanded carrier screening study	23,453	108	417 variants	Current known disease-causing variants only	24%	0.55%	Subjects were referred from obstetric, infertility, and genetic clinics and would likely not be entirely representative of the majority of couples
Picci et al. (2010)	A 10-year CF carrier screening study	59,782	1	47 variants	The 47 most common CF causing variants within Italy.	3.2%	0.4%	78% of carriers did not have a prior family history of CF
								Subjects undergoing MAR had significantly higher CF carrier rate compared with non-MAR subjects
Abouelhoda et al. (2016)	Exome sequencing to estimate carrier frequencies	1549	42	357 genes sequenced (618 variants identified)	Exome sequencing then subsequent alignment and variant calling within specific genes	21.4%	N/A	58% of disease-causing variants were not seen outside the family
Bell et al. (2011)	Carrier testing for severe childhood diseases	104	448	437 genes sequenced (specific variants not targeted)	Sequencing of all coding exons, splice site junctions, and intronic, regulatory, and untranslated regions known to contain disease mutations in 7717 regions from 437 target genes.	97.1%	N/A	No significant differences in total carrier status were found between Caucasians and other ethnicities, suggesting that targeted population testing is likely to be ineffective.
					Interpretation of variants carried out using HGMD			76% of DNA samples were from patients who were known carriers or suffers of AR diseases.
Punj et al. (2018)	Preconception carrier screening by Genome Sequencing	202	728 (gene-disorder pairs)	728 genes sequenced (specific variants not targeted)	Genome sequencing then use of a bioinformatics pipeline for alignment and variant calling. Annotation of variants using multiple sources	78%	16.9%	3% of the subjects were already known to be CF carriers.
Hogan et al. (2018)	Expanded carrier screen via full-exon sequencing and panel-wide copy number variant identification	36,859	234	235 genes sequenced (specific variants not targeted)	Sequencing of coding regions, targeted assessment of pathogenic noncoding variants, panel-wide CNV calling and specialized assays. Customized bioinformatics used to call variants	56%	4.4%	Specific attention given to disease genes with challenging sequence features.
Haque et al. (2016)	Quantifying the modeled risk of recessive conditions identifiable by an expanded carrier screening panel	346,790	94	Genotyped samples- 417 variants. NGS samples- 110 genes sequenced (specific variants not targeted)	Targeted genotyping and NGS with subsequent bioinformatics pipeline for variant calling and annotation.	17%	2%	Individuals with fertility issues or family history of AR conditions were excluded.
								89% of samples processed by targeted genotyping.
Beauchamp et al. (2019a, 2019b)	Clinical impact and cost-effectiveness of a 176-condition expanded carrier screen	66,036	176	176 genes sequenced	Sequencing of coding regions, targeted assessment of pathogenic noncoding variants, panel-wide CNV calling and specialized assays. Customized bioinformatics used to call variants	N/A	1.2%	Individuals with fertility issues or family history of AR conditions were excluded.

Inclusion criteria: (1) studies looking at carrier screening (particularly expanded panels) and reporting on the specifics of the gene/variant panels and the frequency of carriers within the participant group, (2) publication date 2008–present, (3) all geographical locations, (4) English language. Outcome measures: gene/variant panels and frequency of carriers

The American College of Obstetricians and Gynaecologists (ACOG) issued a committee opinion in 2017 advising that only conditions with a carrier frequency of > 1% should be screened for on an expanded panel (Box 2) (ACOG 2017). A study by Guo et al. used gnomAD data to calculate carrier frequency and predicted couple carrier frequencies for 15,795 variants in 415 genes (Guo and Gregg 2019). Strikingly, they found that screening solely for conditions with carrier frequencies of > 1% based on gnomAD data (Karczewski et al. 2019), which equated to variants in just 40 genes, would identify 76–97% of carrier couples (Guo and Gregg 2019). This suggests that the inclusion of additional genes may not balance the additional costs and potential harms of uninformed decision-making and over-diagnosis, particularly around conditions with variable penetrance, onset, and prognosis. The highest couple carrier frequencies were 2.5% and 1.9%, where both individuals were of Ashkenazi Jewish or African ancestry, respectively (Guo and Gregg 2019). All other intra-ancestry couples were found to have couple carrier frequencies of < 1% and inter-ancestry carrier couples frequencies were found to be as low as 0.17% (Guo and Gregg 2019). Another data-driven study found restricting the panel only to those conditions with > 1% carrier frequencies in any ethnicity missed only 11% of carrier couples when compared with a 176-gene panel inclusive of conditions with < 1% carrier frequency (Ben-Shachar et al. 2019). This study also recognised that detection of at-risk couples also saturates, despite the addition of large numbers of additional rare conditions (Ben-Shachar et al. 2019).

**Table:** Box 2 A summary of current guidelines for expanded carrier screening

Summary of the European Society of Human Genetics (ESHG) recommendations for the responsible implementation of expanded carrier screening (ECS) in 2016.	
1. The primary objective of carrier screening should be to enable autonomous choices.	
2. Panels should include a comprehensive set of severe childhood-onset disorders with clear clinical significance. Tests should achieve high clinical validity.	
3. An evidence base should be established and continuously developed and solidified while screening takes place.	
4. ECS should ideally be offered preconceptionally as this maximises reproductive options and has fewer time constraints.	
5. The effectiveness of ECS programmes should be measured by assessing the extent to which it optimises informed choice and reproductive decision making. Not by demonstrating how much it reduces the birth prevalence of affected children.	
6. Attention should be given to psychological, social and counselling-related aspects of ECS.	
7. Couples should be adequately informed at the pre- test stage about the goals, concepts and implications of carrier screening.	
8. ECS should be by voluntary participation.	
9. Genetic testing, information and counselling should be provided by accredited genetic services and appropriately trained professionals.	
10. It should be made explicit to those receiving ECS that care will continue to be provided to them regardless of their reproductive choices.	
11. Health care professionals involved in the provision of ECS should receive appropriate education and training.	
12. Governments and public health authorities should adopt an active role in developing an implementation plan, ensuring quality control and promoting equity of access.	
Summary of the American College of Obstetricians and Gynaecologists (ACOG) committee opinion 690 in 2017 on carrier screening.	
1. Ethnic-specific, pan-ethnic, and ECS are acceptable strategies for pre-pregnancy and prenatal carrier screening.	
2. All patients who are considering pregnancy or are already pregnant, regardless of screening strategy and ethnicity, should be offered carrier screening for cystic fibrosis and spinal muscular atrophy, as well as a complete blood count and screening for thalassemias and hemoglobinopathies. Additional screening also may be indicated based on family history or specific ethnicity.	
3. Carrier screening will not identify all individuals who are at risk of the screened conditions. Patients should be counselled regarding residual risk with any test result.	
4. Prenatal carrier screening does not replace newborn screening.	
5. If a woman is found to be a carrier for a specific condition, her reproductive partner should be offered screening to provide accurate genetic counselling for the couple with regard to the risk of having an affected child.	
6. If a carrier couple is identified before pregnancy, genetic counselling is encouraged so that reproductive options (e.g. donor gametes, preimplantation genetic diagnosis, prenatal diagnosis) can be discussed.	
7. Individuals with a family history of a genetic disorder may benefit from the identification of the specific familial mutation or mutations rather than carrier screening.	
8. Conditions included in ECS panels should: have a carrier frequency of 1 in 100 or greater, have a well-defined phenotype, have a detrimental effect on quality of life, cause cognitive or physical impairment, require surgical or medical intervention, or have an onset early in life. Conditions should be able to be diagnosed prenatally and may afford opportunities for antenatal intervention to improve perinatal outcomes.	
9. Carrier screening panels should not include conditions primarily associated with a disease of adult onset.	

### Gene panel inclusion criteria

**Fig. 3:**
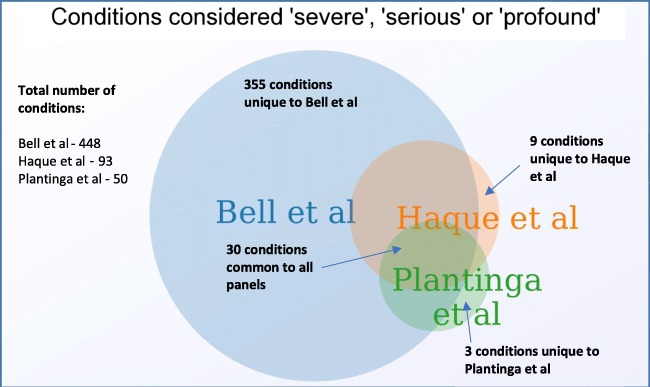
A Venn diagram of three studies which declared conditions included in their panels were ‘severe’, ‘serious’, or ‘profound’. Data from: Bell et al. (2011), Haque et al. (2016), and Plantinga et al. (2016) (These three studies were selected as the classification by condition was readily available as a supplement to their manuscripts, thus enabling the construction of the Venn diagram)

EUCS panel condition inclusion criteria vary significantly with some including mild or adult-onset conditions (Lazarin et al. 2013; Punj et al. 2018). Bell et al. screened for 448 ‘severe’ X-linked and AR conditions (Bell et al. 2011), including primary coenzyme Q10 deficiency, which can sometimes be so mild as to present when a patient is > 60 years (Genetics Home Reference). Three large studies (Bell et al. 2011; Haque et al. 2016; Plantinga et al. 2016) that explicitly chose panel composition based on conditions being ‘severe’, ‘serious’, or ‘profound’ show wide variability with surprisingly few conditions overlapping all three panels (Fig. 3). A recent study compared 16 different providers of EUCS panels and found the number of conditions varied from 41 to 1792 with only three conditions (CF, maple syrup urine disease 1b, and Niemann–Pick disease) shared by all providers (Chokoshvili et al. 2018). Where the same gene was screened, there were substantial differences in the variants included, interpretation and reporting strategies (Chokoshvili et al. 2018).

The inclusion of large numbers of conditions and of those with variable severity raises ethical issues and makes it challenging to provide adequate pre- and post-test education to couples (Ioannou et al. 2010), which is paramount in facilitating informed decision-making. A taxonomy has been proposed by Korngiebel et al. to help with these dilemmas (Korngiebel et al. 2016), whereby categories of conditions could be used as a guide for decision-making and choosing what to screen for. Categories could include severity, treatability, likely life expectancy, and age of onset. These could help simplify the decision process for couples deciding on which conditions they would like to be screened for. Korngiebel et al. used qualitative focus groups of research teams and then patient opinions to design the taxonomy (Korngiebel et al. 2016). The difficulty in defining taxonomy categories that were meaningful to patients as well as clinicians was highlighted, and the frequent re-categorisation of conditions that are variable and unpredictable in their course (Korngiebel et al. 2016). If taxonomy were to be used as part of an EUCS programme, it would need to be standardised and require regular reviews and updates.

### Genes versus variants

Studies exploring EUCS have used different methods of testing for AR conditions and specific causative variants (Table 4). These vary from using allele-specific testing such as genotyping arrays for a limited number of variants associated with a single AR disease gene (Picci et al. 2010), to GWS to test all variants found in any number of AR genes (Punj et al. 2018). A targeted approach simplifies variant interpretation and avoids variants of uncertain significance (VUS) but risks missing carriers if only a limited number of causative variants are genotyped (Stafler et al. 2016; Beauchamp et al. 2019a) and would require regular reviewing and updating. Additionally, there is wide variability between laboratories regarding which variants to report (Hoffman et al. 2014), potentially resulting in substantial health disparities. In contrast, GWS could potentially identify all known variants within a list of reportable genes (Punj et al. 2018), including copy number variants (CNVs) and those that are ultra-rare or private to specific populations with high rates of consanguineous marriages (Abouelhoda et al. 2016; Antonarakis 2019). Punj et al. reported that of 304 variants detected in 134 AR genes in 202 individuals, 14% of all variants were novel (Punj et al. 2018). Despite guidelines on the interpretation of variants (Richards et al. 2015), novel and some rare variants present significant interpretation challenges and there is no consensus on how to handle VUSs. Moreover, many variants in databases of disease genes are erroneously associated with disease (Biesecker 2012) and discordant assertions of pathogenicity between different laboratories are only now starting to be rectified (Harrison et al. 2017). To facilitate reproductive autonomy and limit the undesirable impact of VUSs when making imminent reproductive decisions, a balance would need to be struck between open disclosure and selective reporting. Moreover, even with the increasing utilisation of GWS and continual improvement in variant interpretation, no test will entirely eliminate the risk of having a child affected by an AR condition.

### Implementation

The primary aim of a EUCS programme funded by the healthcare service needs to be clearly identified, as this will influence delivery of the programme. The European Society of Human Genetics (ESHG) published guidelines on the responsible implementation of an EUCS programme in 2016 (see Box 2), stating that the primary objective of a programme should be increased reproductive autonomy; efficacy should thus be assessed by the extent to which the programme optimises informed choice (Henneman et al. 2016). However, many of the empirical evaluations of carrier screening to date have assessed the change in birth frequency (Table 2) rather than attempting to directly measure changes in informed choice. Whilst reduced prevalence of affected children is a likely consequence of increased reproductive autonomy, the ESHG is clear that this should not be used as an evaluation outcome for EUCS (Henneman et al. 2016).

Increased reproductive autonomy is a difficult outcome to assess and make accurate estimates on the cost-effectiveness of the programme. Using solely a reduction in birth frequency as the primary outcome, recent decision-tree modelling has suggested that a 176-condition EUCS test could be close to being cost-effective (based on $50,000 per life year) if 77% of carrier couples chose to prevent an affected birth (Beauchamp et al. 2019b). This threshold would not meet current National Institute for Health and Care Excellence (NICE) recommendations (between £20,000 and £30,000 per quality adjusted life years) (National Institute for Health and Care Excellence and NHS England 2016) and therefore would not be deemed cost-effective for implementation in the UK. Costs of implementation need not only to encompass running the test, but also the extra time and staff needed to provide adequate education, consent, and counselling which is paramount to support reproductive decision-making.

**Fig. 4:**
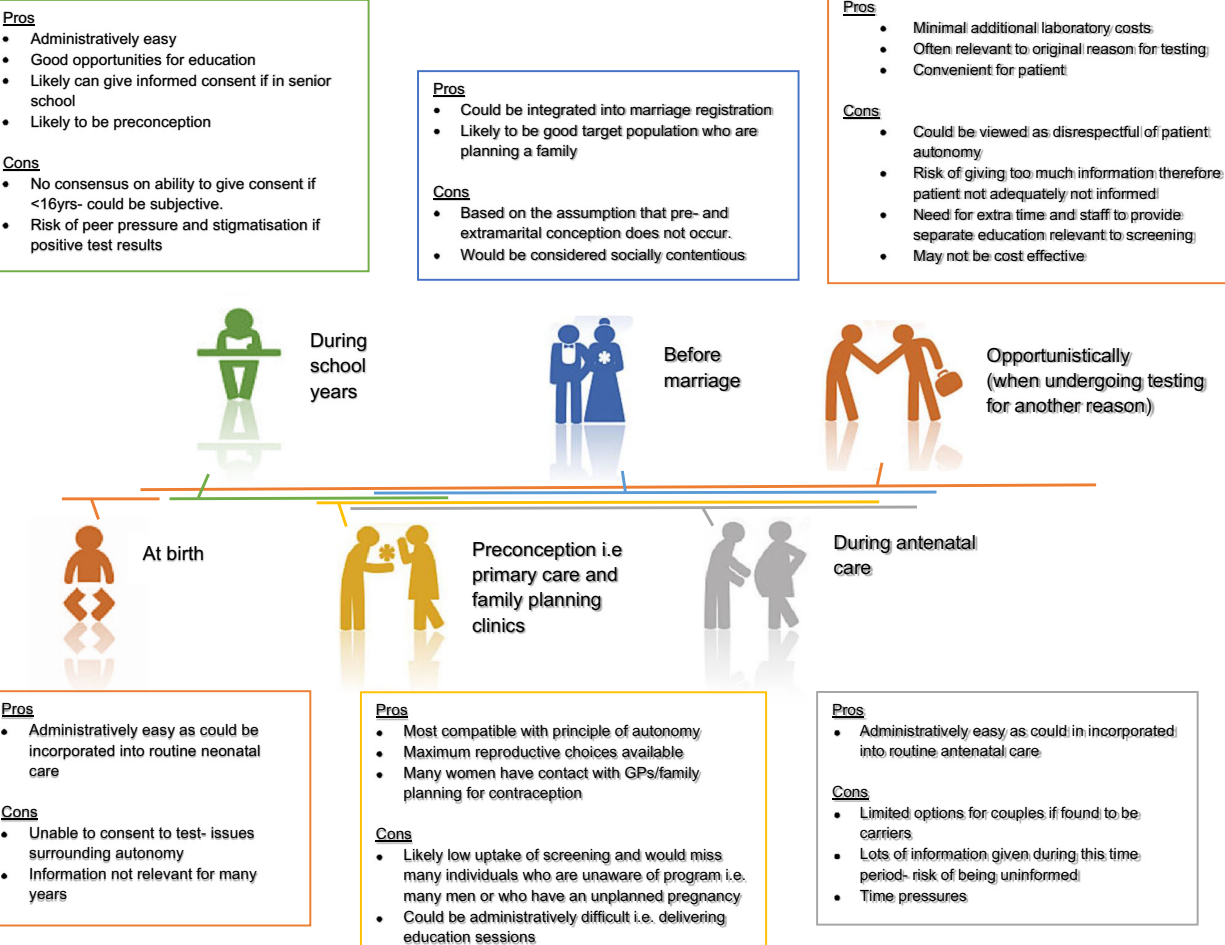
An overview of the pros and cons of carrier screening at different opportunities during an individual’s life (relevant to reproduction)

When and where EUCS could occur needs careful consideration, as it will influence cost, uptake, and the reproductive options available to couples (Fig. 4 and Box 3) (Human Genetics Commission April 2011). A number of implementation strategies have been trialled to date which are aligned with the culture, religion, and ethnic groups represented (Table 2). Programmes which offer premarital or preconception screening tend to have a higher reduction in affected births (Scriver et al. 1984; Mitchell et al. 1996). In multi-cultural societies such as the UK, standardisation of an EUCSprogramme would likely be challenging. Details of implementation would also need to consider which health care professionals should offer the service, their educational needs, and the knock-on resource implications for the healthcare service. Future training of existing healthcare professionals and the likely need to employ new personnel to deliver the programme will be a substantial initial and ongoing expenditure.

**Table:** Box 3 Reproductive options available to carrier couples during the preconception period compared with when pregnancy is established (antenatal). Information from Human Genetics Commission, Increasing options, informing choice: A report on preconception genetic testing and screening; April 2011

**Reproductive options available to carrier couples before conception**	**Reproductive options to carrier couples after pregnancy is established**
Remain childless Adopt a child	Undergo prenatal diagnosis to establish whether the foetus is affected; this information can then be used to decide whether to terminate or continue the pregnancy
	Accept the chance of having an affected child
Accept the chance of having an affected child.	
Conceive naturally then undergo prenatal diagnosis to establish whether the foetus is affected; this information can then be used to decide whether to terminate or continue the pregnancy	
Conceive using donated sperm or eggs	
Undergo in vitro fertilisation to allow preimplantation genetic diagnosis of embryos and transfer unaffected embryos to the woman to begin a pregnancy.

#### At birth or during school years

Offering EUCS at birth or in school years would be administratively relatively easy, as screening could align with other tests or vaccinations and education could be given to large groups during school. Since it would be preconception, such a programme would also allow individuals to know their carrier status before considering starting a family. One study evaluating a single-gene carrier screening programme for senior school age children in Australia found knowledge was good, there was minimal concern about carrier status, no stigma was experienced, and there was a high level of satisfaction with the programme overall (Barlow-Stewart et al. 2003). However, the challenge of providing adequate education for EUCS has raised questions whether consent would be sufficiently informed (Ioannou et al. 2010). Ethical arguments against genetic testing in babies and young children include respecting their future autonomy, potential psychological or social harm to the child, and harm from lack of disclosure of results by the parents, but evidence of actual harm being caused is limited (Vears and Metcalfe 2015).

#### Preconception/premarital

In the UK, free contraception is widely available, and many women access this through GP surgeries and family planning clinics during their reproductive years. This presents an opportunity to offer EUCS to individuals who are already taking control of their reproductive autonomy. However, nearly 20% of pregnancies are unplanned (Wellings et al. 2013) and so many individuals may miss the opportunity. Similarly, whilst testing during the premarital period has been successful in some cultures (Cowan 2009), in the UK, 48% of births occur outside of marriage or civil partnership (Haine 2017). In vitro fertilisation (IVF) clinics could offer EUCS—albeit to a limited number of individuals—and indeed, some already do (The Centre for Reproductive & Genetic Health, 2019). Couples within IVF clinics are already proactive in making reproductive decisions and offering carrier screening may provide additional information that could increase the chances of a healthy offspring by embryo selection.

#### Antenatally

Carrier screening during antenatal care is already undertaken in the UK for beta-thalassaemia and sickle cell anaemia so the process of expanding this to a universally applicable panel would be administratively easy. However, it has been argued that increasing the number of antenatal tests could negatively impact on couples’ ability to give informed consent (Beard et al. 2016). The timing of the test is also crucial to maximise reproductive options, and pressure to complete testing within an actionable timeframe is stressful (Beard et al. 2016).

#### Opportunistically

Opportunistic screening constitutes actively investigating variants unrelated to the primary clinical indication for the test but relating to secondary indications, such as carrier status (Wright et al. 2011). Offering EUCS alongside clinically indicated GWS would be a logistically simple approach. The UK 100,000 Genomes Project offers CF carrier screening as part of their additional (secondary) findings (The 100,000 Genomes Project 2019) and in the USA, the ACMG have recommended four AR and X-linked conditions that should be reported following clinical GWS regardless of the initial clinical indication (Kalia et al. 2017). Whilst the cost implications for opportunistic screening of this kind are minimal, it provides poor equity of access.

#### Individuals versus couples

When screening for large numbers of conditions, including the ultra-rare, it is likely that an individual will be positive for carrying at least one AR condition (Srinivasan et al. 2010). However, the chance of a couple being joint carriers for the same condition is very low (Haque et al. 2016; Guo and Gregg 2019). Screening women could occur in the first instance with partners only undergoing screening if she was found to be a carrier. The second stage screening could be targeted, and potentially save costs. However, in time pressured situations, this method may not be suitable and would not be applicable if the couple were to have children with different partners in the future.

### Social and ethical considerations

The social and ethical implications of carrier screening have been extensively discussed (Buchanan et al. 2000). The dominant view is that carrier screening enhances reproductive autonomy (De Wert et al. 2012; van der Hout et al. 2019) and has potential to prevent suffering of children with genetic conditions (Plantinga et al. 2016; De Wert et al. 2012). However, carrier screening encroaches on ethical and cultural values; therefore, individuals will vary in their views of what is acceptable to screen for. There is concern that, despite reproductive autonomy being the primary objective, any routinisation of carrier screening could alter social norms and place new societal pressure on couples to make particular choices (Kihlbom 2016). Screening could potentially be perceived as taking a discriminatory view of those already living with specific conditions, particularly where effective treatments exist (Tom Shakespeare 2019), such as with CF. It could also lead to stigmatisation of couples who decline screening and subsequently give birth to affected children. Attitudes towards carrier screening from individuals living with genetic diseases that could be prevented through carrier screening are often favourable, but vary based on prior personal experience of the condition (Boardman et al. 2018).

There have been several studies exploring public views on carrier screening (Table 5) which have offered insight into potential implementation strategies. Overall views are generally positive towards carrier screening, particularly for single-gene tests (Fu et al. 2016; McClaren et al. 2008) and in those who already have good knowledge about carrier status and risk (Plantinga et al. 2016). General themes of underestimating risk and a need for more education were common themes (Plantinga et al. 2016; Beard et al. 2016; Ekstrand Ragnar et al. 2016; Fu et al. 2016; McClaren et al. 2008). There were higher levels of uncertainty when multiple conditions were included (Plantinga et al. 2016; Mathijssen et al. 2018), or when no condition-specific information was given (Ekstrand Ragnar et al. 2016). Many felt screening for multiple conditions was convenient (Beard et al. 2016) but, unsurprisingly, knowledge decreased (Ioannou et al. 2010). Alarmingly, when participants of one study were asked about the presence of genetic conditions in their family, most reported multifactorial diseases such as diabetes and heart disease (Gilmore et al. 2017), suggesting a low level of understanding about carrier screening and raising concerns about informed consent. Better education and prior knowledge increase uptake of screening and reduce stress (Scott et al. 2010; Shao et al. 2015).

**Table 5:** A comparison of public opinion studies of various universal carrier screening tests. *PC* preconception, *A* antenatal, *PN* postnatal, *PCS* preconception carrier screening, *CF* cystic fibrosis, *SMA* spinal muscular atrophy, *FXS* Fragile X syndrome, *TSD* Tay-Sachs disease, *FAC* Fanconi anaemia type C, *BS* Bloom Syndrome, *CD* Canavan disease, *NPA* Neimann-Pick disease type A, and *FD* familial dysautonomia

Study	Carrier screen	Method	Outcomes	Limitations
Fu et al. (2016)	PCS for AR deafness	Quantitative, hypothetical study	• 98.5% underestimated their risk of being a carrier for AR deafness.	Nonresponse bias
				Only for one condition- not representative of an expanded carrier screening test
		975 individuals sampled from 2 colleges (including staff).	• 66.9% would be willing to have a genetic test for carrier status of AR deafness	
China				
		Questionnaires. Information included in questionnaire and not given beforehand		
McClaren et al. (2008)	Carrier screening for CF	Quantitative and qualitative, hypothetical study	• Most participants supported universal CF carrier screening	Nonresponse bias
		68 participants (individuals and couples preconception and antenatally)	• Attitudes were influenced by their current knowledge/experience.	Only for CF
Australia				
		Questionnaire on paper, focus groups and interviews.	• Supportive of preconception testing	32% of participants were people with a family history of CF
		Nil prior education on CF		
Plantinga et al. (2016)	PCS for 70 genes associated with 50 very serious, untreatable, early onset AR disease	Quantitative, hypothetical study	• 34% would take the test if offered	Imbalance of male/female respondents (72% female)
		504 individuals (preconception) aged 18–40 recruited by a survey research sampling company.	• 15% would be unlikely to take it	
			• 51% were still undecided	
			• Majority (44%) would prefer the test to be offered by the GP	Nonresponse bias
Netherlands				
		Questionnaires. Information provided before the questionnaire	• Majority (37%) preferred face-to-face consultation to gain information	
Ekstrand Ragnar et al. (2016)	PCS (conditions not specified)	Quantitative, hypothetical study	• 32% were interested in PCS	Nonresponse bias
		777 men/women partners filled out 3 questionnaires (early pregnancy, at 34 weeks and 12 months after delivery)	• 27% were not interested	No specific carrier screen was discussed, i.e. single-gene or expanded panel
Sweden				
		Information included in questionnaire and not given beforehand	• 41% were uncertain	
Schuurmans et al. (2019)	PCS for 50 severe conditions	Quantitative study	• Test acceptors more frequently had a higher education level	Nonresponse bias
		questionnai190 couples interested in the PCS filled in res before and after testing (or declining test)	• ‘Sparing a child a life with a severe condition’ was the most common reason to have testing	
Netherlands				
			• ‘The test-result would not influence their decision to have children’ was the most important reason for declining the test	
Beard et al. (2016)	Carrier screening for CF, SMA, and FXS	Qualitative study	• All women appreciated the convenience of undergoing screening for 3 conditions simultaneously	Nonresponse bias
		10 women who had undergone expanded carrier screening (8 were pregnant at the time of testing) and who were found to be carriers of CF, SMA, or FXS were interviewed.	• All women supported universal screening	Bias of women who were positive for a AR condition
Australia			• All but one felt the best time would be before preconception	No men included in the study
			• Highest levels of anxiety were whilst waiting for the partners test results in couples who were already pregnant as the main concern was the prospect of considering a termination.	
Gilmore et al. (2017)	PCS for 750 AR, X-linked and mitochondrial conditions and ~ 100 medically actionable incidental findings	Quantitative study	The most common reasons for declining were:	No men included in the study
		240 women (who had already undergone CF carrier screening) declined a PCS carrier screen completed a questionnaire about their reasons for declining.	• Time or travel limitations	Nonresponse bias
			• Lack of interest/not wanting to know the information	
USA				
			• Anxiety/worry	
			‘Late decliners’ (declined after receiving the consent form) were more likely to report ‘do not want to know’ or ‘anxiety/worry’	Not assessing opinions of those who had not already had CF screening
Ioannou et al. (2010)	Carrier screening for TSD, CF, FAC, BS, CD NPA, and FD in Ashkenazi Jewish high schools	Qualitative study	• 74.1% found the screening to be convenient	Not representative of the general population as Ashkenazi Jewish population tend to be very supportive of screening
		272 students (ages 15–17 years) who had been offered expanded carrier screening completed a questionnaire about their experience.	• 87.1% felt they had enough prior information to make a decision about screening	
Australia			• Uptake of screening was 99.6%	
		Information was given before screening via face-to-face presentation, a DVD and brochures.	• Knowledge was lower and anxiety levels higher for the expanded panel when compared with single-gene screening	
Mathijssen et al. (2018)	Carrier screening for PCH2, FADS, rhizomelic chrondrodysplasia punctate type 1 and osteogenesis imperfecta in a Dutch Founder population	Quantitative study	• 97% did not regret testing	Not representative of the general population as Dutch Founder populations tend to have higher knowledge of AR conditions and are supportive of screening.
		182 participants who accepted the offer of carrier screening (preconception and antenatal) completed a pre and post-test questionnaire.	• 97% would recommend it to others	
			• 94% stated pretest counselling should be offered	
Netherlands		137 non-attendees also completed a separate questionnaire	• 100% made reproductive decisions based on the results	
			• Main reason for non-attendance was being unaware of the programme	

Inclusion criteria: (1) studies looking at public opinion on carrier screening including both hypothetical studies and those where testing was carried out, (2) publication date 2008–present, (3) all geographical locations, (4) English language. Outcome measures: general opinions, reasons for declining carrier screening, opinions of timing/location, education, and counselling. Commentary articles written to convey opinion/discussion with no research component where excluded

Carrier couples reported the result would definitely affect their reproductive decision-making (Mathijssen et al. 2018). Opinions were overwhelmingly in favour of testing in the preconception period as opposed to antenatally (McClaren et al. 2008; Beard et al. 2016), a finding primarily driven by women experiencing high levels of anxiety whilst having screening during their pregnancy (Beard et al. 2016) and by those who already had a child with CF and would have liked the opportunity to utilise preconception options (McClaren et al. 2008). Amongst those who were uninterested or unsure about carrier screening, reasons were largely ‘not wanting to know’ and disagreeing with the selection of children based on genetic tests (Plantinga et al. 2016; Beard et al. 2016; Ekstrand Ragnar et al. 2016). A recent Dutch study of offering expanded carrier screening to couples from the general population found that “sparing a child a life with a severe genetic condition was the most important reason to accept”, whilst “the most important reason for declining was that the test-result would not affect participants’ reproductive decisions” (Schuurmans et al. 2019).

### For-profit and direct-to-consumer (DTC) testing

There are a number of companies offering for-profit and/or DTC carrier screening, and these have been a major driver in the promotion of EUCS panels. Some companies have generated a large proportion of the recent research data in this area (Beauchamp et al. 2019b; Lazarin et al. 2013; Srinivasan et al. 2010). It has been argued that DTC testing empowers individuals to access and utilise their genomic data; however, there are concerns that consumers lack adequate education and support when receiving and interpreting results and therefore may be at risk of harm (Chokoshvili et al. 2017). Regulation of DTC testing varies significantly between countries and types of tests, with some imposing restrictions or requiring medical supervision (Kalokairinou et al. 2018). Some DTC tests can only be ordered by a healthcare professional (‘physician-mediated’) and the consumer is thereby supported during the testing process (Chokoshvili et al. 2017) but the cost of testing is still met by the consumer. Costs accrued for counselling and follow-up would likely fall upon the healthcare service, which could be considerable even when DTC testing is undertaken without initial physician support. Moreover, DTC testing is limited to those who can afford it, thus excluding lower socio-economic groups.

## Conclusions

Whilst carrier screening is a well-established practice, it has been slow to take advantage of the new possibilities offered by rapidly developing genomic technology. Evidence from previous screening programmes, expanded carrier screening studies, and public opinion demonstrate the potential benefits of offering an EUCS programme. However, the overall chance of a non-consanguineous couple being ‘at-risk’ of having a child with an AR condition is very low and the primary outcome of increased reproductive autonomy will be challenging to measure. This makes it difficult to provide evidence that investing in EUCS will be more beneficial than other healthcare interventions.

Many issues have been identified and discussed as requiring careful consideration by a government wanting to implement EUCS (Molster et al. 2017). The use of GWS for multiple AR diseases covering all ethnicities is attractive in order to achieve maximum sensitivity; however, the inclusion of numerous conditions and variants complicates the screening process. It could not only cause harm (Ioannou et al. 2010), but could render the test less cost-effective as risk reduction for extremely rare conditions is minimal (Haque et al. 2016). Social and ethical issues are closely linked with the overall aims of the programme, choice of which conditions to include, timing of the test, and moral acceptability of reproductive options available to individuals. The best compromise could be that conditions universally screened for should be relatively common, have a high carrier frequency, be serious enough to significantly impact the affected individual and their family, and benefit from the availability of an accurate, sensitive test.

Much of the research to date has either been on population-specific, ancestry-based carrier screening or, if on EUCS, has been driven by commercial companies, neither of which are representative of a publicly funded EUCS programme. A group in the Netherlands are in the process of piloting a preconception, EUCS via GPs (Plantinga et al. 2016). The results of which will hopefully be beneficial in evaluating whether EUCS is possible, desirable, and cost-effective within a public healthcare system. However, until more representative data can be obtained, it is difficult to justify creating a publicly funded EUCS programme based on the current evidence.
